# Selective Targeting of Protein Arginine Methyltransferase 1 (PRMT1) Mutant Activity by a Small Molecule Activation Strategy

**DOI:** 10.1021/acschembio.6c00073

**Published:** 2026-04-28

**Authors:** Tran Dang, Y. George Zheng

**Affiliations:** Department of Pharmaceutical and Biomedical Sciences, College of Pharmacy, 1355 University of Georgia, Athens, Georgia 30602, United States

## Abstract

Protein arginine methyltransferase 1 (PRMT1) is involved in the post-translational modification of cellular proteins in higher organisms by adding a methyl group to arginine-containing substrates. Various studies have demonstrated that the overexpression of PRMT1 is common in cancer development and correlates with poor survival prognosis of patients. Selective targeting of individual PRMT isoforms with chemical inhibitors is technically challenging due to the high sequence similarity among them. This study aims to create a distinct method to selectively modulate PRMT1 activity with small molecules. In our approach, enzymatically inactive PRMT1 forms were produced by mutating residue H293 to alanine or glycine by site-directed mutagenesis. Next, an exogenous chemical molecule was used to activate the enzymatic activity of the silent PRMT1 form by introducing a structurally complementary functional group at the mutation site. We demonstrated that the small molecule compound 4-methylimidazole effectively enhanced PRMT1-H293G activity by 8.6-fold while not affecting the enzymatic activities of the other PRMT isoforms. The selective enhancement effect of small molecule activation of PRMT1 was observed on both single-substrate and complex proteome mixtures. Hence, we presented a unique chemical genetics strategy to modulate PRMT1 activity with small molecules with high isoform selectivity, which can be of great potential to be applied to dissect cellular functions of individual particular PRMT members with fast onset and in a real-time manner.

## Graphical abstract

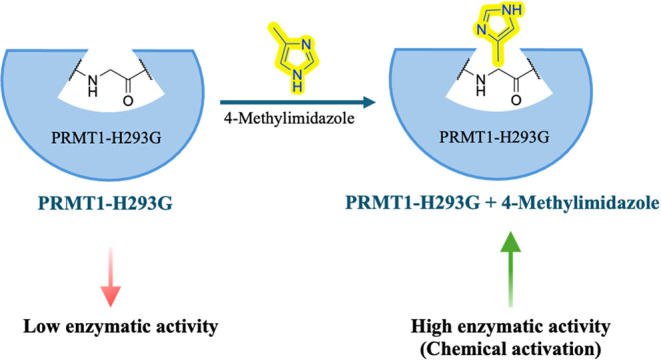

## Introduction

Protein arginine methyltransferases (PRMTs) play a major role in the post-translational modification of cellular proteins in eukaryotic organisms. There are nine members in the PRMT family in human cells, which are grouped into three different types.^1^ All PRMTs use S-adenosyl-l-methionine (SAM) as a methyl donor to methylate the side-chain guanidino group of arginine residues. Type-I PRMTs consisted of PRMT1, 2, 3, 4 (CARM1 (co-activator-associated arginine methyltransferase 1)), 6, and 8.^2–7^ Type I produces asymmetric dimethylarginine (ADMA) on the same guanidino group of the substrate. Conversely, type-II PRMTs, PRMT5 and PRMT9, methylate the adjacent guanidino group to create symmetrical dimethylarginine (SDMA).^8,9^ PRMT7 is the only type-III PRMT, which only monomethylates its substrate to create monomethyl arginine (MMA).^10^

PRMT1 is the predominant type-I PRMT member that regulates and maintains multiple cellular processes such as β-cell development, gene transcription, and signal transduction.^11–13^ A notable substrate of PRMT1 is the histone H4 N-terminal tail at the arginine-3 residue site.^14,15^ The knockout of PRMT1 is lethal in mice.^16,17^ Furthermore, PRMT1 dysregulation is observed in many cancers and other diseases.^18–23^ The overexpression of PRMT1 correlates with a high mortality rate in cancer patients.^23,24^ As a result, extensive research is conducted on developing small molecule inhibitors of PRMT1 and other PRMT members for therapeutic application.^25–27^ Small molecule inhibitors are also of great merit as pharmacological probes to dissect the functional roles of particular PRMTs in disease pathways. Some outstanding inhibitors for type-I PRMTs are MS023, GSK3368715, DB75, and K313. MS023 inhibits PRMTs in different pathways, such as impaired RNA splicing and histone methylation.^28,29^ GSK3368715 strongly diminishes the ADMA level, and its combination treatment with GSK3326595 can greatly shrink the tumor volume.^27^ Diamidine inhibitors such as furamidine (DB75) and K313 have similar inhibitory mechanisms against PRMT1 and were shown to block leukemia cell growth and reduce RBM15 methylation.^25,30^ Nevertheless, a common major weakness of thus far developed PRMT1 inhibitors is a lack of isoform selectivity.^31,32^ This is somehow expected, as the sequence homology among type-I PRMT members is high, and small molecule probes inhibiting PRMT1 activity often have off-target effects on the other type-I PRMT isoforms as well.

To advance the research of developing and applying small molecule chemical tools to selectively target enzymatic activities of a particular PRMT isoform, in this work, we developed a chemical activation method to specifically modulate the enzymatic activity of PRMT1. In this approach, a key histidine residue in the active site of PRMT1 is mutated to glycine, creating a catalytically defective enzyme form (Figure 1). Then, the activity of the PRMT1 mutant is restored by small molecules, such as imidazole or its analogs that substitute for the missing side-chain structure of the mutated residue. We conducted a suite of biochemical assays to test and validate the activity activation of the PRMT1 mutant by small molecules. Impressively, the rescue ligand acts selectively on the mutant PRMT1 form with no interfering effect on the wild-type PRMT1 (PRMT1-WT) and other PRMT isoforms. This is the first chemical activation study using a small molecule to selectively target the enzymatic activity of a particular type-I PRMT isoform.

**Figure 1:**
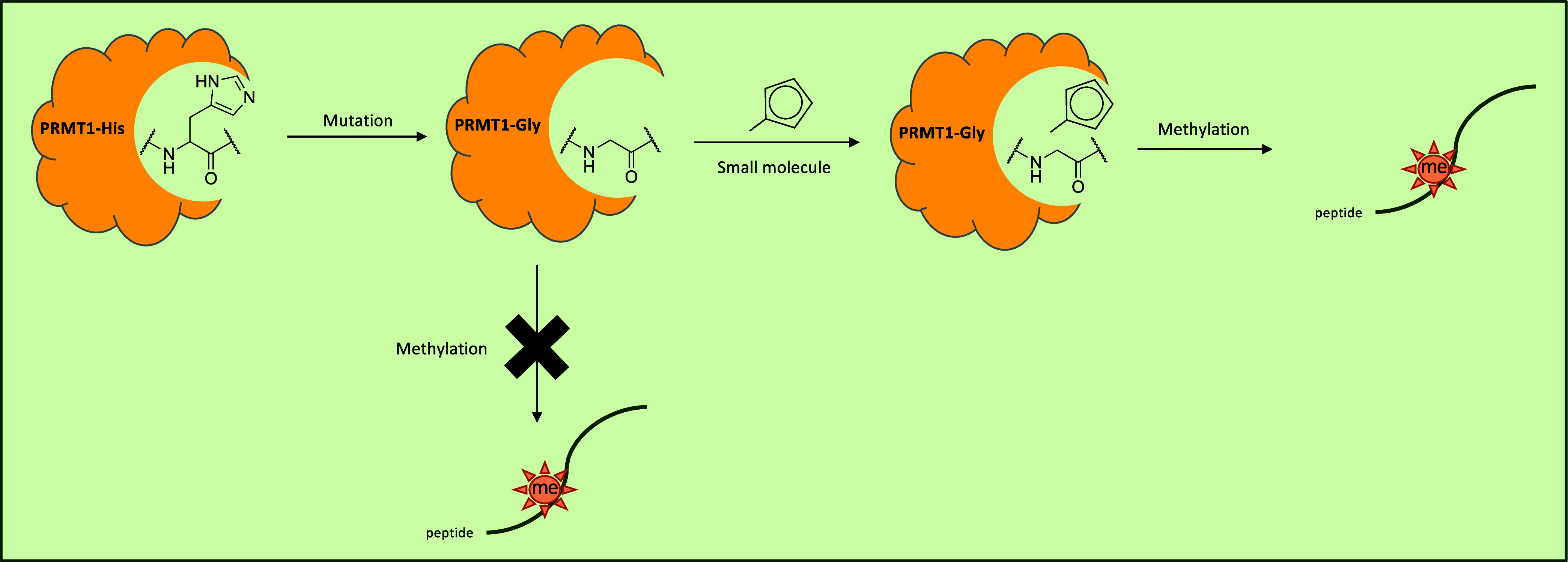
Chemical rescue scheme of PRMT1-H293G. A histidine residue at position 293 was mutated to glycine, and a small molecule subsequently activated the methyltransferase activity.

## Results

### Activity Comparison of PRMT1-WT versus PRMT1 Mutants

To develop a chemical biology strategy for the selective modulation of PRMT1 activity with small molecules, we chose to mutate the residue histidine 293 in PRMT1. Histidine 293 is located on the THW loop of PRMT1 in the active site and is essential for the catalytic activity of PRMT1. Indeed, a previous study showed that the *k*_cat_/*K*_M_ of the PRMT1-H293A mutant exhibited a decrease of 256-fold of activity, possibly owing to the disruption of the salt bridge formed between H293 and D51.^33^ Another study demonstrated that H293 is involved in substrate orientation and product specificity.^34^

We performed site-directed mutagenesis on the H293 residue of PRMT1, mutating this site to either glycine or alanine using the QuikChange site-directed mutagenesis by PCR.^35^ The DNA plasmid encodes the PRMT1 catalytic domain (11–353) in the pET28b vector. Protein expressions of PRMT1 wild-type (PRMT1-WT) and PRMT1 mutants were achieved in *Escherichia coli* BL21­(DE3). The expressed PRMT1 proteins have a 6xHis-tag on the *N*-terminal end. Therefore, the proteins were purified by Immobilized Metal Chelate Affinity Chromatography (IMAC), using Nickel-NTA agarose resin beads. The protein was desalted by a HiTrap desalting column prepacked with a Sephadex G-25 resin to remove any traces of salts that could interfere with the protein activation experiment. The protein was loaded on sodium dodecyl sulfate-polyacrylamide gel electrophoresis (SDS-PAGE) to confirm the expected size for downstream experiments.

Next, we measured the methyltransferase activities of PRMT1 and PRMT1 mutants using the scintillation proximity assay (SPA) that we established earlier.^36,37^ The enzymatic reaction mixture consisted of the enzyme, [^3^H]-SAM, and peptide H4(1–20)-Biotin (sequence: SG**R**­GKG­GKG­LGK­GGAK­RHRK-(Biotin)), incubated for 30 min at RT, and then quenched by adding isopropanol in an equal volume. Subsequently, 10 μL of 20 mg mL^–1^ streptavidin-coated SPA beads were added to the mixture, and scintillation counting was performed on a MicroBeta 2450 Microplate Counter to obtain the count per min (CPM). As shown in Figure 2, the single-point activities of PRMT1-H293A and PRMT1-H293G were significantly lower than PRMT1-WT, 59-fold and 25-fold, respectively. This result demonstrated that the activities of the mutants were near the background level and suitable for further experiments.

**Figure 2:**
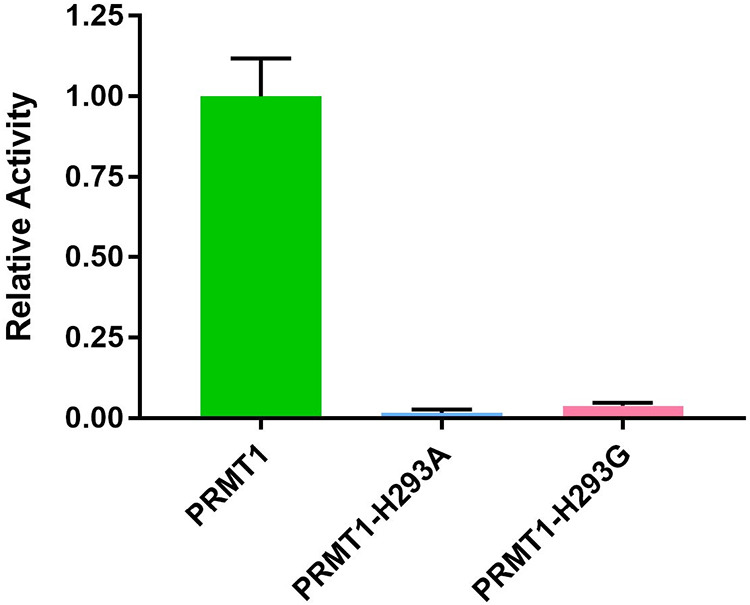
Single-point methyltransferase activity determination of PRMT1-WT and PRMT1 mutants. In vitro activity screening comprised 0.02 μM enzyme, 0.5 μM radioactive [^3^H]-SAM, and 1 μM of H4(1–20)-Biotin peptide. The reaction was quenched with 100% isopropanol after 30 min. Each sample was performed in duplicate.

### Chemical Activation of PRMT1 Mutants with Small Molecule Compounds

Mutation of H293 to alanine or glycine resulted in a “hole” in PRMT1, as the side-chain imidazole group of the residue was lost. We projected that chemical molecules with similar structures and sizes of the missing imidazole side chain could potentially fit in the “hole” to complement the missing side-chain imidazole group and thus restore the enzymatic activity of PRMT1-H293 mutants.^38,39^ Some of such restoration molecules for histidine to alanine and glycine mutation can be imidazole analogs.^40,41^ We chose several compounds: imidazole, 2-methylimidazole, 4-methylimidazole, 2-ethylimidazole, and 1*H*-1,2,3-Triazole to test their ability to rescue the activity of the PRMT1-H293A or PRMT1-H293G mutant (Figure 3A). A single-point methyltransferase activity test was performed to examine small molecules for their rescue activities. Most literature has suggested that rescue experiments be done at millimolar concentrations of rescue compounds.^41,42^ Therefore, 20 mM of each compound was chosen for the experiment. The reaction mixture contained 0.02 μM enzyme, 1 μM H4(1–20)-Biotin peptide, and 0.5 μM [^3^H]-SAM, with individual rescue compounds, respectively, and for an incubation time of 30 min. The methylation activities are shown in Figure 3B. None of these exogenous compounds was found to significantly rescue the methyltransferase activity of PRMT1-H293A. By contrast, the activity of PRMT1-H293G was successfully activated by imidazole and 4-methylimidazole, with 2.3-fold and 4.8-fold activation, respectively (Figure 3C). Our data are consistent with the previous notion that the glycine mutants are usually rescued much more efficiently than the alanine mutants.^43^ Based on these results, our following tests will be focused on the chemical activation of PRMT1-H293G by 4-methylimidazole.

**Figure 3:**
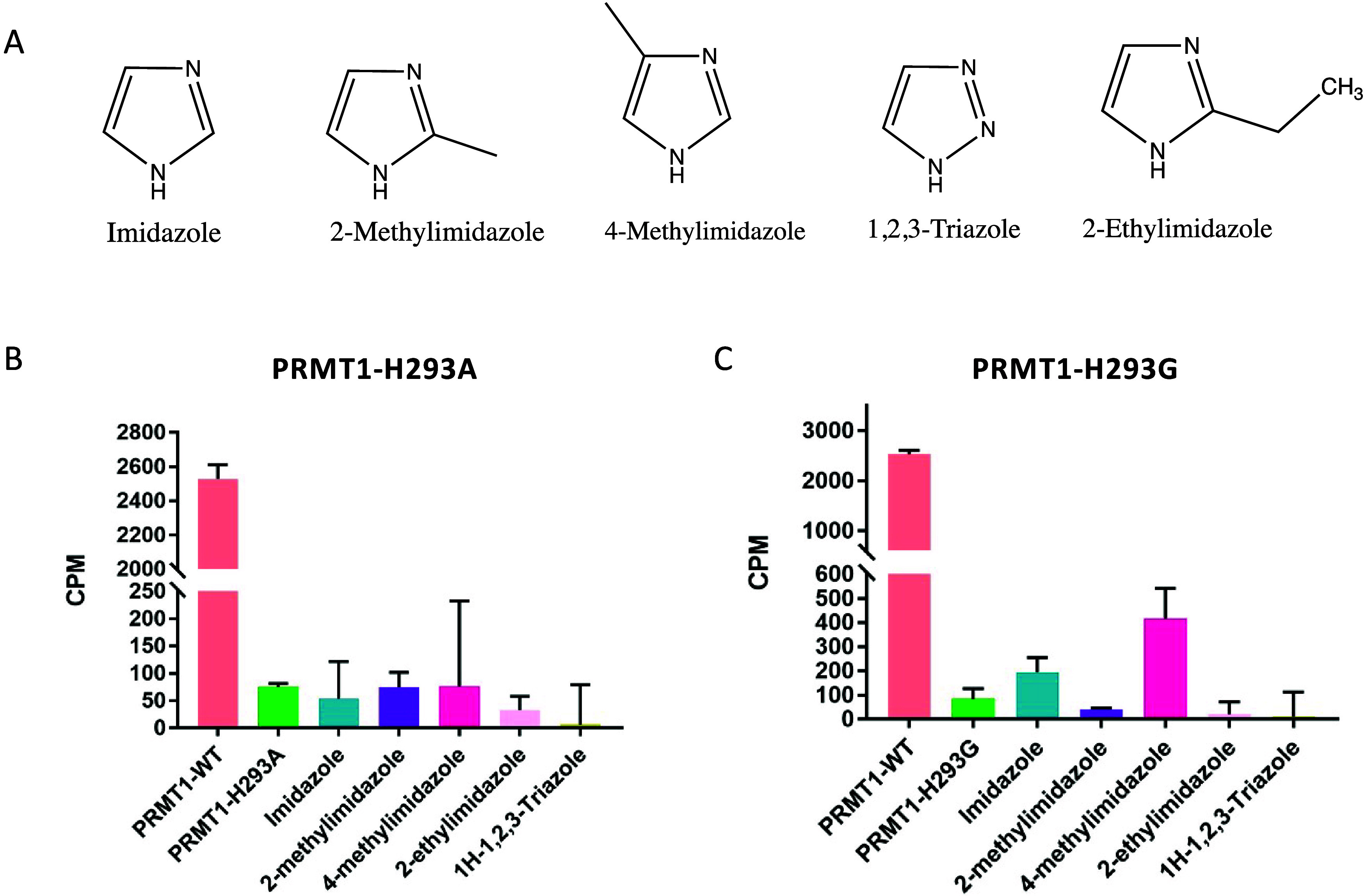
Activating PRMT1 mutants by exogenous molecules. (A) small molecules chosen for this study. (B and C) PRMT1-H293A and PRMT1-H293G were activated by different compounds. The concentration of each tested exogenous compound was 20 mM. The screening reaction comprised an exogenous molecule, 0.02 μM of enzyme, 0.5 μM of [^3^H]-SAM, and 1 μM of H4(1–20)-Biotin peptide. The reaction time was 30 min, and the reaction was quenched with 100% isopropanol. Each sample was performed in duplicate.

### Concentration Dependence of PRMT1-H293G Activation by 4-Methylimidazole

To characterize the activity restoration behavior controlled by the protein–ligand interaction, it would be crucial to determine the ideal concentration of 4-methylimidazole to rescue the activity of PRMT1-H293G. Chemical activation experiments often required tens to hundreds of millimolar concentrations of rescue molecules.^42,44^ Therefore, various concentrations from 0.5 to 64 mM of 4-methylimidazole were tested using the same experimental protocol as mentioned in the above section. The result clearly showed a concentration-dependent activation of PRMT1-H293G activity by 4-methylimidazole, and 20–40 mM 4-methylimidazole provided the best rescue activity (Figure 4). The enzyme activity of PRMT1-H293G decreased a bit as the concentration of 4-methylimidazole exceeded 64 mM, which was likely due to the saturation of the vacant histidine-to-glycine mutation site by 4-methylimidazole, as well as some minor side effects of the compound at high concentrations. Based on these results, we chose 20 mM 4-methylimidazole for further studies.

**Figure 4:**
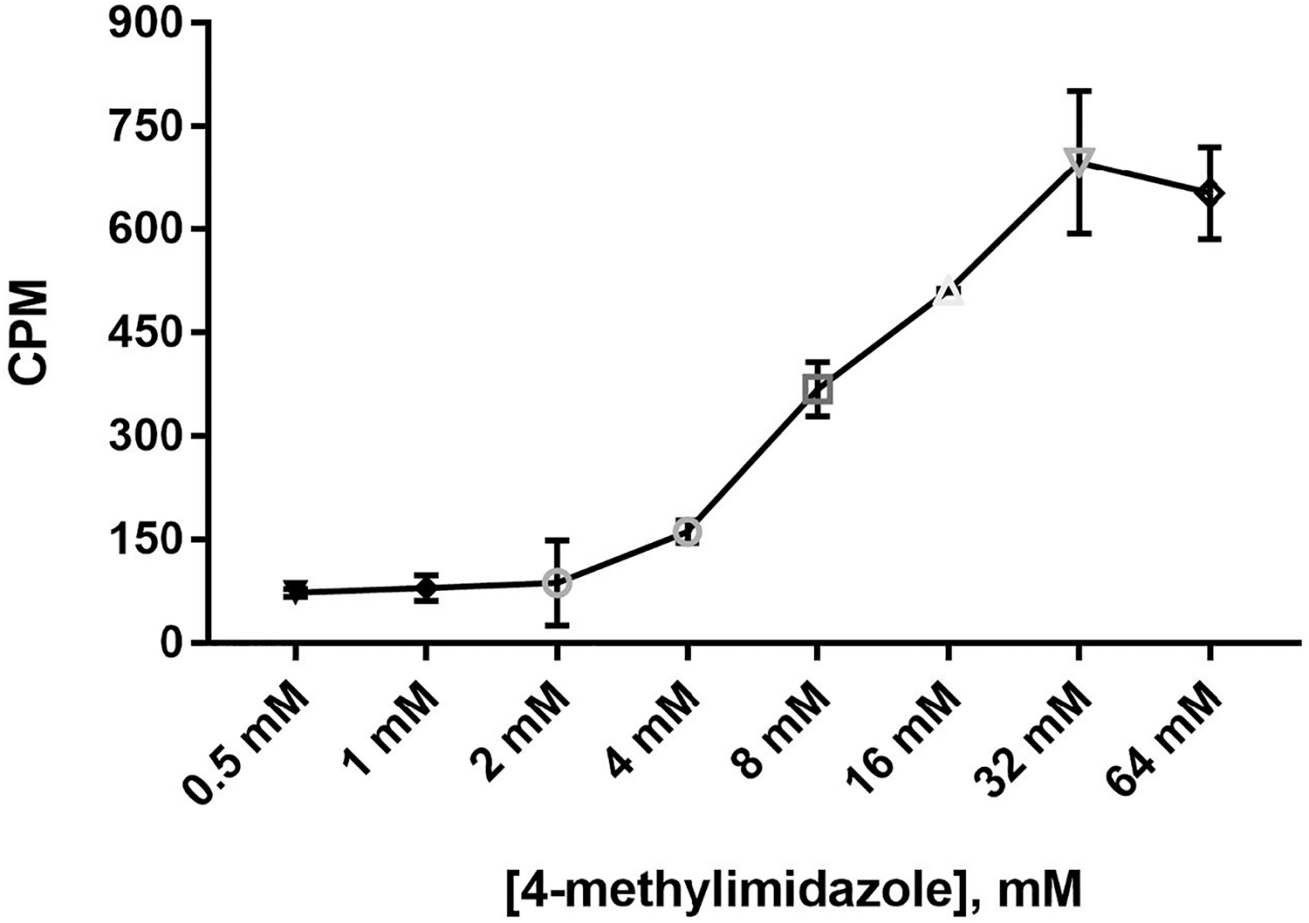
Activating PRMT1-H293G activity by varying the 4-methylimidazole concentration. Various concentrations from 0.5 to 256 mM of 4-methylimidazole, 0.04 μM enzyme, 0.5 μM [^3^H]-SAM, and 1 μM Biotin-H4(1–22) peptide were included in the reaction. The reaction was quenched with 100% isopropanol after 30 min at RT. Each sample was performed in duplicate.

### Effect of 4-Methylimidazole on the Activities of PRMT1-WT and PRMT1-H293G

We were interested in analyzing whether 4-methylimidazole had any effect on PRMT1-WT and to what extent PRMT1-H293G could be rescued at the 20 mM concentration. The reaction consisted of the enzyme, [^3^H]-SAM, and Biotin-H4(1–22) peptide (Biotin-SG**R**­GKG­GKG­LGK­GGA­KRH­RKVL). The activator, 4-methylimidazole, was added to the reaction mixture as needed. The reaction was quenched with isopropanol after 30 min of reaction, and streptavidin-coated SPA beads were added for methylation reading. As displayed in Figure 5, the activity of PRMT1-WT in the presence of 20 mM 4-methylimidazole was comparable to that without activated PRMT1-WT, which proved that 4-methylimidazole did not interfere with the activity of PRMT1-WT. In contrast, 20 mM 4-methylimidazole was able to rescue the activity of PRMT1-H293G by 8.6-fold. This result clearly demonstrated that 4-methylimidazole was specific for PRMT1-H293G, with little interference with the activity of PRMT1-WT.

**Figure 5:**
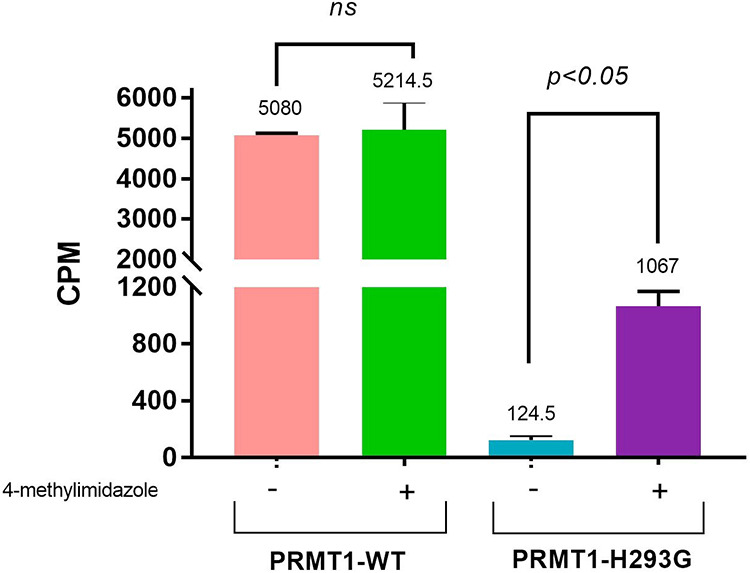
Effect of 4-methylimidazole on PRMT1-WT and PRMT1-H293G. The final concentration of 20 mM of 4-methylimidazole, 0.04 μM enzyme, 0.5 μM [^3^H]-SAM, and 1 μM Biotin-H4(1–22) for reactivating the enzyme. For the negative control without the activator, 4-methylimidazole was not added. The reaction was halted after 30 min with 100% isopropanol. Each sample was performed in duplicate. Statistical analysis was performed using a two-tailed distribution unpaired *t*-test.

### Evaluation of the Activity Activation of PRMT1-H293G by 4-Methylimidazole with Mass Spectrometry

To further confirm the success of activating PRMT1-H293G by 4-methylimidazole, we did an in vitro methylation assay with the use of regular, nonradioactive SAM and analyzed the methylation products of the reaction mixture using matrix-assisted laser desorption/ionization mass spectrometry (MALDI-MS). We used the Ac–H4(1–21) peptide (Ac-SG**R**­GKG­GKG­LGK­GGA­KRH­RKV) as the substrate. It is noteworthy that this peptide could be methylated twice on the arginine-3 residue by PRMT1. The reaction time was 2 h at 25 °C and quenched with 5% TFA. A negative control, consisting only of the Ac–H4(1–21) peptide and SAM, did not show any methylated peptide formation (Figure 6A). As expected, no methylation was seen after the addition of 4-methylimidazole to this solution, since it lacks enzyme presence (Figure 6B). Next, we examined the activity of PRMT1-H293G without the addition of the activating compound. The data showed that PRMT1-H293G was largely inactive, as only a very weak peak of the monomethylated Ac–H4(1–21) peptide was observed (Figure 6C). To demonstrate chemical activation efficacy, 4-methylimidazole was added to the reaction mixture containing SAM, Ac–H4(1–21), and PRMT1-H293G. As expected, the methylated product peaks were significantly higher, with both monomethylated and dimethylated peptides observed as a result of chemical activation by 4-methylimidazole (Figure 6D). In conclusion, PRMT1-H293G regained significant methyltransferase activity after being activated by 4-methylimidazole.

**Figure 6:**
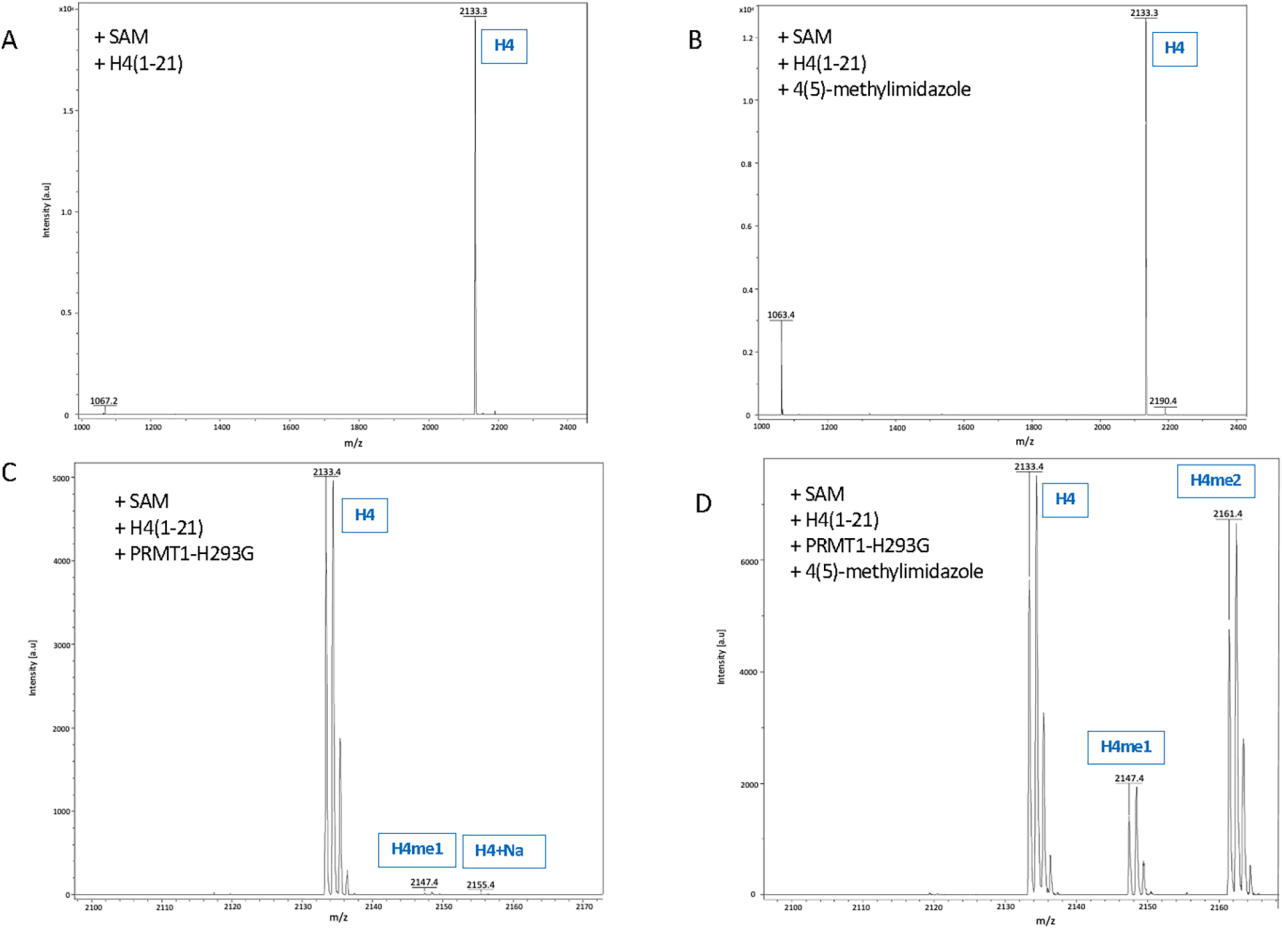
Methylation of the H4(1–21) peptide by activated PRMT1-H293G. (A) No presence of PRMT1-H293G and 4-methylimidazole. (B) No presence of PRMT1-H293G. (C) No presence of 4-methylimidazole. (D) Presence of SAM, PRMT1-H293G, and 4-methylimidazole, and H4(1–21) peptide. PRMT1-H293G, 1 μM, was incubated on ice with 20 mM 4-methylimidazole for 30 min. Subsequently, 10 μM Ac–H4(1–21) and 50 μM SAM were added to the reaction for 2 h at 25 °C. The reaction was quenched with 5% TFA and subjected to MALDI-MS analysis.

We wanted to further confirm the chemical activation behavior of 4-methylimidazole on PRMT1-H293G by using a different peptide substrate. For this purpose, we created a synthetic peptide R4 (Ac-GG**R**­GGF­GG**R**­GGK­GG**R**­GGF­GG**R**­GGFG) that consisted of four arginine sites, which entailed eight possible methylation states. In the presence of only SAM and R4 peptides, only the 2223.341 peptide peak was prominent (Figure 7A). Next, we added 4-methylimidazole to the reaction, and the data did not show any methylation peaks; only the peptide peak was observed, which confirmed that the enzyme had no methyltransferase activity without the presence of the enzyme (Figure 7B). When the PRMT1-H293G protein was added to the SAM and R4 mixture, a weak monomethylation peak was seen in the MS spectrum (Figure 7C). Once again, this same result was similar to that of the H4(1–21) peptide reaction, concluding that PRMT1-H293G still contained a very low background level of methyltransferase activity. On the other hand, when the reaction solution consisted of SAM, R4, PRMT1-H293G, and 4-methylimidazole, multiple methylation states of the R4 substrate, i.e., R4me1, R4me2, R4me3, R4me4, and R4me5, were present (Figure 7D). Clearly, 4-methylimidazole significantly activated the enzymatic activity of PRMT-H193G in peptide substrate methylation.

**Figure 7:**
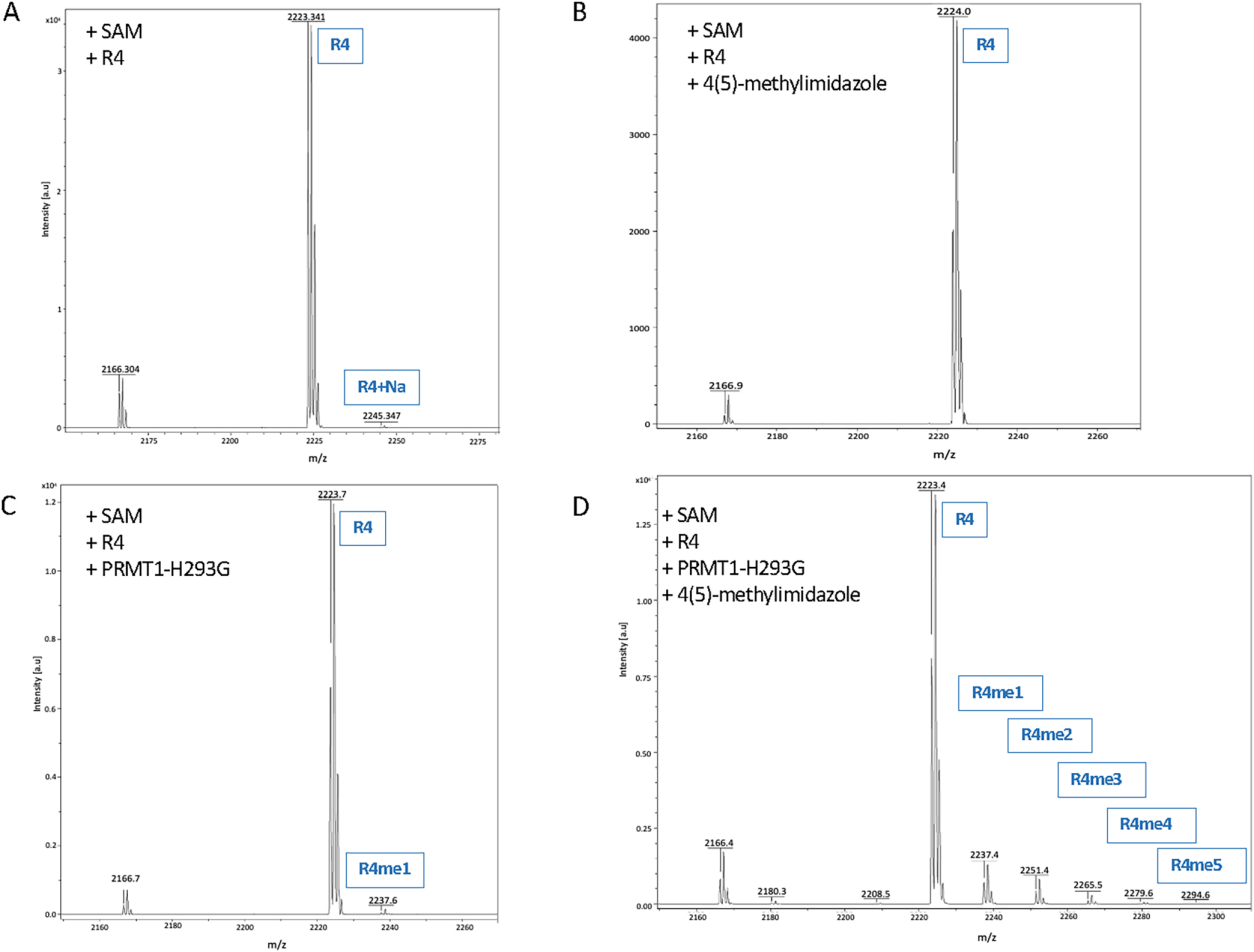
Methylation of the R4 peptide by activated PRMT1-H293G. (A) No PRMT1-H293G and 4-methylimidazole in the reaction mixture. (B) Absence of PRMT1-H293G. (C) Without 4-methylimidazole. (D) In the presence of SAM, PRMT1-H293G, 4-methylimidazole, and R4 peptide. PRMT1-H293G and 4-methylimidazole were equilibrated on ice for 30 min at concentrations of 0.5 μM and 20 mM, respectively. Then, 5 μM R4 and 50 μM SAM were added to the reaction for 30 min at 25 °C. The reaction was quenched with TFA and sent to MALDI-MS.

### Evaluation of the Chemical Activation with Enzyme Kinetics Measurements

We then measured the steady-state kinetic parameters of PRMT1-H293G in the presence or absence of 4-methylimidazole, in order to further understand the chemical activation behavior of 4-methylimidazole. The radiometric methyltransferase assay was used for this biochemical examination. PRMT1-WT or PRMT1-H293G was incubated with 4-methylimidazole in the reaction buffer. Subsequently, various concentrations of Biotin-H4(1–22) (Biotin-SG**R**­GKG­GKG­LGK­GGA­KRH­RKVL) and 0.5 μM [^3^H]-SAM were added to the reaction. After 30 min, the reaction was quenched with isopropanol. The streptavidin-coated SPA beads were added before the methylation signals were read on the MicroBeta instrument. As shown in Figure 8, the PRMT1-H293G mutant showed a very low catalytic activity compared with PRMT1-WT: *k*_cat_ and *k*_cat_/*K*_M_ of PRMT1-H293G were 33-fold (0.002 ± 0.0002 min^–1^) and 42-fold (0.005 min^–1^μM^–1^) lower than that of PRMT1-WT, respectively. These data were in good agreement with the previous reports.^33,34^ Most notably, after the addition of 4-methylimidazole, the *k*_cat_ and *k*_cat_/*K*_M_ of activated PRMT1-H293G increased 18-fold (0.037 ± 0.004 min^–1^) and 19-fold (0.095 min^–1^μM^–1^) compared to those before the activation. The *k*_cat_/*K*_M_ of rescued PRMT1-H293G reached 45% activity of PRMT1-WT. In comparison and as expected, 4-methylimidazole had little effect on the activity of PRMT1-WT. The *K*_M_ values for all of the samples were similar at around 0.3 μM. In combination, the data demonstrated that the chemical activation by this chemical rescue ligand was efficient and very specific for the PRMT1-H293G form.

**Figure 8:**
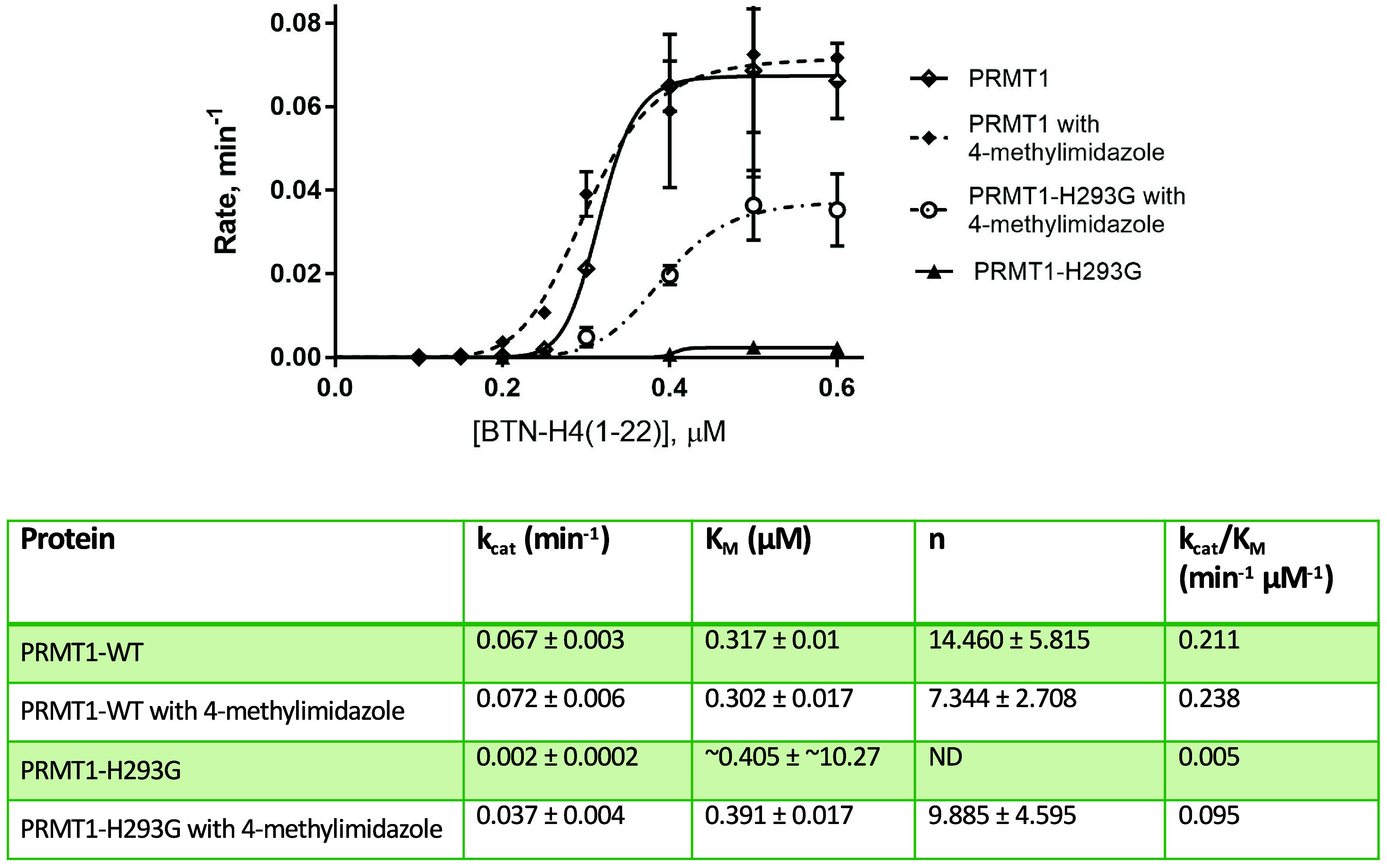
Enzyme kinetics measurement of PRMT1-WT and PRMT1-H293G and activation by 4-methylimidazole. The reaction comprised 20 mM 4-methylimidazole, 0.04 μM enzyme, 0.5 μM [^3^H]-SAM, and varying concentrations of the Biotin-H4(1–22) peptide. Isopropanol was used to quench the reaction after 30 min. ND indicated that the data cannot be determined due to the lack of detectable activity. Each sample was performed in duplicate.

### pH Dependence of the Chemical Activation

The p*K*_a_ of 4-methylimidazole is ∼7.5.^45^ Therefore, at the pH conditions we used, 4-methylimidazole exists as a mixture of the neutral form and the protonated methylimidazolium ion form. To determine which form of 4-methylimidazole assisted in the chemical rescue, we tested the activation of PRMT1-H293G by 4-methylimidazole at several pH values: 6.5, 7.0, 7.5, 8.0, 8.5, and 9.0. PRMT1-H293G, Biotin-H4(1–22) (Biotin-SG**R**­GKG­GKG­LGK­GGA­KRH­RKVL), 4-methylimidazole, and [^3^H]-SAM were mixed in the respective pH buffers. The reaction was conducted at RT for 30 min and quenched with 100% isopropanol. The CPM was detected on the MicroBeta counter after the streptavidin-coated SPA beads were added to the reaction solution. As displayed in Figure 9, the methylation activities of both PRMT1-H293G and activated PRMT1-H293G increased in a pH-dependent manner. At a low pH value of 6.5, the activities of both free PRMT1-H293G and rescued PRMT1-H293G were undetectable. The methylation activity started to show up at pH values higher than 7.0 for both free PRMT1-H293G and activated PRMT1-H293G in the solution (Figure 9). The activity enhancements by 4-methylimidazole at pH 7.5, 8.0, and 8.5 were 1.3-, 2.6-, and 4.3-fold, respectively. These data demonstrated that the chemical activation by 4-methylimidazole is pH-dependent, and higher pH leads to a stronger activation effect. This was suggestive that the deprotonated form of 4-methylimidazole was responsible for the activation of PRMT1-H293G activity. The methylation activities of PRMT1-H293G started to slightly decrease at pH 9.0, which was consistent with the previous studies on pH effects on PRMT1 activity, and most likely the protein started to be denatured under strong basic conditions.^33,46,47^

**Figure 9:**
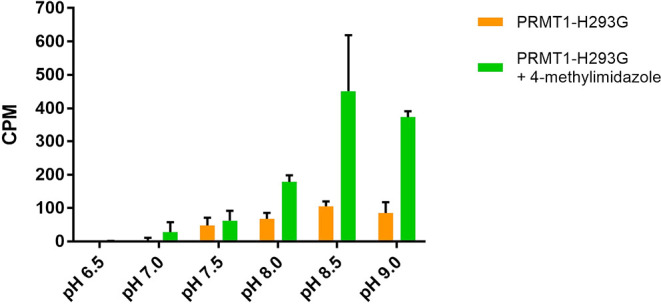
pH effect in activating PRMT1-H293G. PRMT1-H293G and activated PRMT-H293G at various pH levels. All reagents were mixed in each respective pH level with 0.04 μM enzyme, 20 mM 4-methylimidazole, 0.5 μM Biotin-H4(1–22), and 0.5 μM [^3^H]-SAM. The reaction was incubated for 30 min at RT and quenched with isopropanol. Each sample was performed in duplicate.

### Methylation of the HeLa Cell Proteome

We showed that 4-methylimidazole rescued the enzymatic activity of PRMT1-H293G to methylate H4 and R4 peptides in vitro. We were curious whether activated PRMT1-H293G can methylate the complex proteomes of cultured cells. Toward this end, we treated the HeLa cells with 20 μM adenosine dialdehyde (AdOx) for 24 h to globally decrease methylation levels in the cell’s proteome.^48,49^ After cell lysis, the hypomethylated HeLa cell lysate was incubated with PRMT1-H293G or PRMT1-WT protein together with 4-methylimidazole at various concentrations on ice for 30 min before SAM was added to the reaction for 3 more hours at 30 °C. The reaction was quenched with the protein loading buffer and boiled for 10 min at 95 °C. Next, Western blotting was applied to detect the ADMA levels in different samples. As expected, PRMT1-H293G increased the ADMA level of the cell lysates in a 4-methylimidazole concentration-dependent manner (Figure 10). The reaction with 20 mM 4-methylimidazole exhibited the highest ADMA level in the presence of PRMT1-H293G and 4-methylimidazole. On the other hand, 4-methylimidazole had no enhancing impact on the methylation activity of PRMT1-WT in this test (data not shown), further supporting that the enzyme activation effect of this compound is specific for the PRMT1-H293G form.

**Figure 10:**
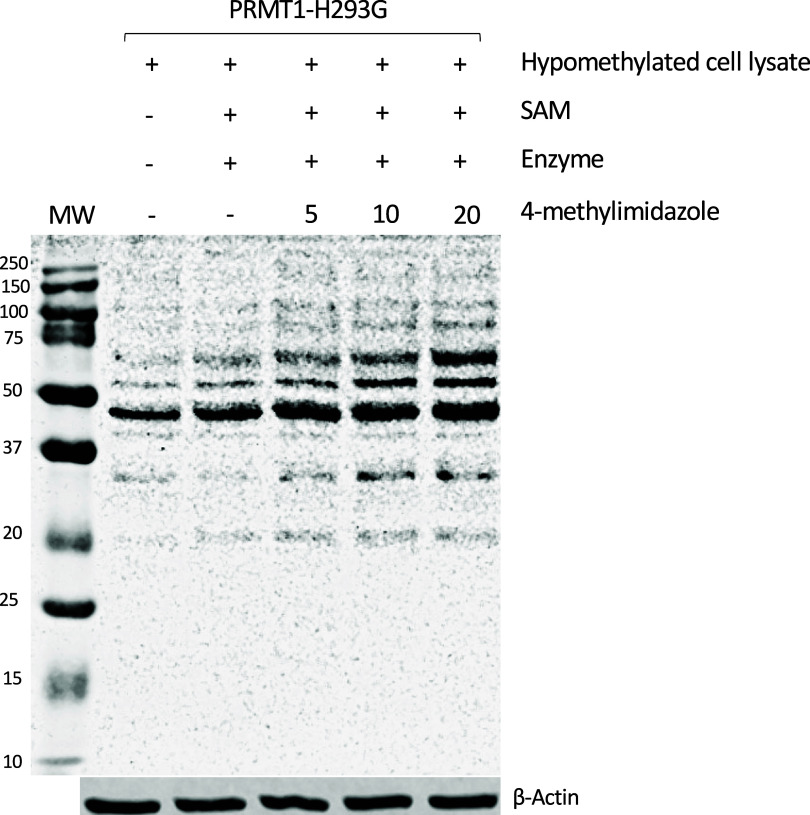
Activated PRMT1-H293G ADMA detection in hypomethylated cell lysate. PRMT1-H293G, 1 μM, was incubated with 5, 10, and 20 mM 4-methylimidazole for 30 min on ice. Subsequently, 15 μg of hypomethylated HeLa cell lysate and 50 μM SAM were added to the reaction for 3 h at 30 °C. The reaction was quenched with 5× loading dye, heated for 10 min at 95 °C, and resolved on SDS-PAGE for Western blotting.

### Testing Effects of 4-Methylimidazole on the Other PRMTs

Our idea was to use 4-methylimidazole as a chemical tool for the selective activation of only the PRMT1-H293G form, without affecting the other PRMTs. Therefore, we tested the interfering effect of 4-methylimidazole on some PRMT enzymes. In this regard, in addition to wild-type PRMT1, the methyltransferase activities of PRMT3 and PRMT8 were tested because both belong to type-I members and can catalyze asymmetrical dimethylation in protein and peptide substrates.^2^ To prevent the off-target effect of 4-methylimidazole on type-II PRMTs, we tested the activation activity of 4-methylimidazole on PRMT5, as it is the major producer of symmetric dimethylarginine.^50^

Radioactive SPA assays were used to measure the activity of PRMT3 and PRMT8. Both enzymes can methylate the Biotin-H4(1–22) peptide (Biotin-SG**R**­GKG­GKG­LGK­GGA­KRH­RKVL) in the presence of [^3^H]-SAM. Separately, 4-methylimidazole was added to examine the difference in methyltransferase activity. The reaction was at RT for 30 min and quenched with isopropanol. Streptavidin-coated SPA beads were added to the reaction of the CPM reading by the MicroBeta instrument. As shown in Figure 11, PRMT3 did not show any significant difference in the presence of 4-methylimidazole. Likewise, 4-methylimidazole had a negligible effect on the PRMT8 activity.

**Figure 11:**
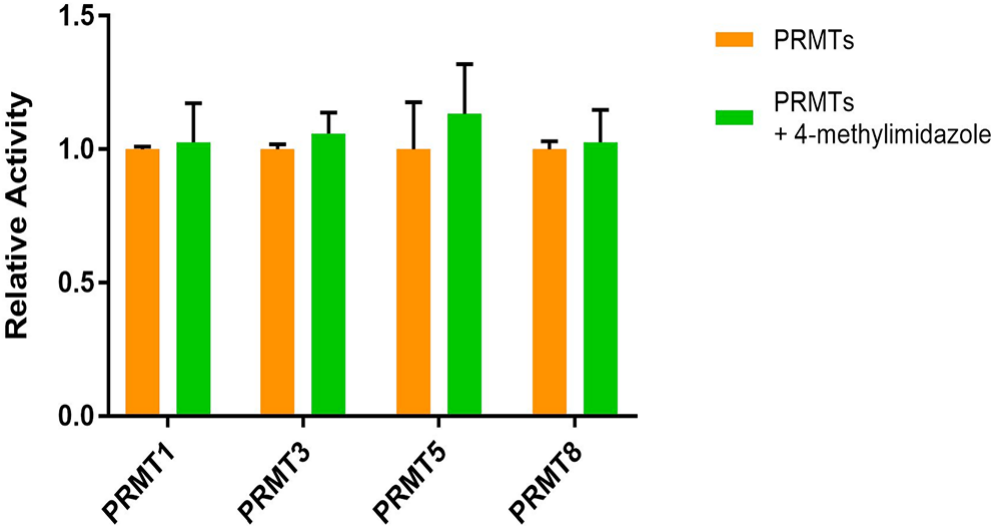
Activation effect of 4-methylimidazole on PRMTs. Relative activity of PRMTs and 4-methylimidazole-activated PRMTs was measured by radioactive assays. Each arginine methyltransferase protein at a concentration of 0.04 μM was mixed with 0.5 μM peptide and 0.5 μM [^3^H]-SAM. For 4-methylimidazole-activated samples, 20 mM 4-methylimidazole was added to the reaction and incubated for 30 min at RT, and the reaction was quenched with 100% isopropanol. Each sample was performed in duplicate.

We used the filter binding radioactive assay (FBA) to measure the activity of PRMT5 and the effect of 4-methylimidazole on PRMT5. The peptide for PRMT5 was Ac–H4(1–21) (Ac-SG**R**­GKG­GKG­LGK­GGA­KRH­RKV). With this method, we transferred the reaction solution onto phosphocellulose P81 paper to air-dry after ending the reaction with isopropanol. After 30 min, the phosphocellulose paper was washed with 50 mM NaHCO3 to remove all the unspecific binding and left to air-dry overnight. The next day, the phosphocellulose paper was inserted in a vial and soaked in a scintillation cocktail. The reading was performed by a Beckman Coulter LS6500. PRMT5 methylated the H4(1–21) (Ac-SG**R**­GKG­GKG­LGK­GGA­KRH­RKV) substrate readily. The data demonstrated that 4-methylimidazole had very little effect on the activity of PRMT5 (Figure 11). Therefore, 4-methylimidazole was only effective for activating the PRMT1-H293G form.

## Discussion

Our work presented in this study explored the strategy of enzyme activation by small molecules to study the functions of PRMTs in biological pathways. The methodology is to revive a catalytically dead enzyme with a structurally similar compound to the missing motif in native proteins.^51,52^ Some of the prominent advantages with using chemical activation over genetic transfection in elucidating the mechanism of a protein include: no requirement for gene knock-in, fast onset time, controlling activity degrees of the enzyme by adjusting amounts of activator agents, and reversibility of the process to control protein function. For instance, chemical activation of enzyme activity by an effector drug is near instantaneous as compared to the lag phase required for maximum induction of protein expression during transfection. Depending on the types of mutation sites, some popular exogenous molecules that are used to activate various enzymes include amines, imidazoles, and indoles.^38,53–57^ Quite a few studies have demonstrated the success of this technique in vitro, such as the activity restoration of the phosphate dehydrogenase mutant by aminoguanidine and the protein tyrosine Src R388A mutant by imidazole.^39,57^ Additionally, protein activation in vivo by small molecules was demonstrated in *E. coli* and *Saccharomyces cerevisiae* and in mammalian cells.^38,55–58^

Histidine residue 293 in PRMT1 is located on the THW loop and is proposed to play a role in binding to the carboxyl backbone of peptide substrates during the arginine methylation process.^59^ Therefore, mutating H293 to alanine and glycine will create a catalytically defective enzyme form.^33^ From a mechanistic viewpoint, the loss of activity of PRMT1-H293A/G, as well as the observation that this mutant revived its activity in the presence of methylimidazole, signifies the importance of the H293 residue for PRMT1 catalysis. PRMT1-H293G is a loss-of-function enzyme that provides an ideal protein model for chemical activation by a chemical genetics approach. This study demonstrated that PRMT1-H293G activity can be significantly increased by 4-methylimidazole. The steady-state kinetics experiments showed that the activity of PRMT1-H293G was significantly lower than that of the wild-type PRMT1 and was able to regain its activity up to 45% by 4-methylimidazole when compared to wild-type PRMT1 (Figure 8). 4-Methylimidazole activated PRMT1-H293G activity more effectively than imidazole, which might be due to its better structural fit into the mutation site: it has a methyl group, making it closely resemble the missing structure of the histidine side chain. Aligning with this structural hypothesis, PRMT1-H293G but not PRMT1-H293A can be activated by the molecules presented in this study. A reason for this outcome might be steric collision with the methyl group of alanine, whereas PRMT1-H293G does not carry a bulky group. Therefore, the structure of alanine could possibly prevent proper binding of small molecules to the vacant pocket. This reveals that fine-tuning the structures of rescue molecules is important to the success in rescuing the mutated residue.

After regaining its activity by 4-methylimidazole, PRMT1-H293G was able to methylate the substrate peptides H4 and R4 (Figures 6 and 7). The study with methylation of the hypomethylated cell lysates supported the idea that the activated PRMT1-H293G can methylate different substrates (Figure 10). We want to point out that tens of millimolar concentrations of a rescue ligand are typically needed for effective activation of an enzyme mutant in chemical rescue studies. Such a requirement of a high concentration of the activating compound can be attributed to multiple factors, such as weak noncovalent binding affinity, small sizes of the compound, dynamic fluctuation of the ligand binding site, and solvent influence. Of importance, overly high concentrations of a rescue compound should be cautioned for chemical activation, which is due to potential further influence on the enzyme structure and activity. For instance, we clearly observed that the activity of PRMT1-H293G started to decrease when the concentrations of 4-methylimidazole increased to 60 mM and beyond (Figure 4). Such a phenomenon was similarly seen in previous SpyCas9 and nucleoside diphosphate kinase chemical rescue studies.^41,42^ One reason for this high-concentration toxicity might be the saturation of the rescue ligands at the binding site, and the presence of extra activators can prevent substrate binding to the active site, which results in reduced enzyme activity. Another reason might be the rescue ligand triggering conformational changes of the protein targets, which is detrimental to their activity. However, these hypotheses require a further detailed mechanistic investigation.

The pH test shows that the activity of PRMT1-H293G increased in a pH-dependent manner, reaching a maximum at pH 8.5 (Figure 9). Neither the enzyme nor 4-methylimidazole functioned under highly acidic conditions. These results indicate that the side-chain imidazole group of the H293 residue assists PRMT1-WT catalysis in its deprotonated state, and, in consistency, 4-methylimidazole activates PRMT1-H293G in the deprotonated form. The off-target effect was one of the main concerns when using small molecules in enzyme modulation. Therefore, we tested 4-methylimidazole against PRMT1-WT and PRMT3, 5, and 8. Both PRMT3 and PRMT8 can asymmetrically dimethylate their substrates like PRMT1. Our results showed no significant changes in activity for PRMT1-WT, PRMT3, and PRMT8 after the addition of 4-methylimidazole (Figure 11). PRMT5 is a major type-II methyltransferase that forms symmetric dimethylarginine (SDMA) in proteins. The data showed that 4-methylimidazole had very little effect on the activity of PRMT5 (Figure 11). Overall, in all of the wild-type PRMTs, no significant interference was observed. Therefore, 4-methylimidazole shows selective and utmost activation with PRMT1-H293G but no off-target effects against other type-I or type-II isoforms.

In conclusion, our small molecule activation of PRMT1 mutant activity demonstrated in this study has proven the methodology used to use small chemical molecules to selectively modulate a particular PRMT isoform. This can be a useful chemical genetic method to elucidate the biological functions of individual PRMT members. The future perspective is to expand the selective chemical activation tool to investigate the enzymatic functions of PRMT1 in particular biological or disease pathways under in vivo contexts. In this regard, the chemical activation approach will be of superior advantage over chemical inhibition, as most inhibitor compounds developed so far fall short of isoform selectivity among different type-I PRMT members. It is notable that PRMTs, including PRMT1, are often found to be mutated in cancer clinical samples (https://portal.gdc.cancer.gov/), and chemical modulation of naturally mutated PRMTs can be a novel avenue of drug development.^60^

## Methods

### Protein Expression and Purification

QuikChange direct-site mutagenesis on PRMT1 histidine 293 was performed for alanine and glycine mutations. The primers were ordered from Integrated DNA Technology (IDT) with standard desalting. The primer sequence of PRMT1-H293A consisted of 5′-GAG­TCT­CCA­TAC­ACA­GCG­AAG­XAG­ACT­GTG-3′ for the forward primer and 5′-CAC­AGT­CTG­CTT­CCA­CGC­TGT­GTA­TGG­AGA­CTC-3′ for the reverse primer. The forward primer for PRMT1-H293G was 5′-GAG­TCT­CCA­TAC­ACA­GGC­TGG­AAG­CAG­ACT­GTG-3′, and the reverse primer sequence was 5′-CAC­AGT­CTG­CTT­CCA­GCC­TGT­GTA­TGG­AGA­CTC-3′. A polymerase chain reaction (PCR) was performed using a 20 ng/mL DNA pET28b-PRMT1 template, 40 ng/mL forward and reverse primers, 10 mM dNTP, native pfu polymerase, and 10× native plus pfu buffer. The PCR product was digested by DpnI at 37 °C for 1 h and loaded onto 1% agarose gel following the PCR. Subsequently, the best PCR product was transformed into *E. coli* DH5α by heat shock and plated on LB agar with kanamycin for overnight incubation at 37 °C. The DNA plasmid was extracted by the Wizard Plus Minipreps DNA Purification System kit (Promega) on the next day and was sent to ACGT Inc. for sequencing.

After the confirmation of the correct mutation, the plasmid was transformed into *E. coli* BL21-CodonPlus­(DE3)-RIL for protein expression. The recombinant PRMT1-WT and PRMT1 mutants were expressed and purified by Ni-NTA as in previous studies.^61^ The protein was further purified by a HiTrap desalting column prepacked with Sephadex G-25 resin (product number: 29048684, GE Healthcare Bio-Sciences) to get rid of salts and small molecules. The protein was eluted in storage buffer containing 25 mM Na-HEPES, pH 7, 300 mM NaCl, 10% glycerol, and 1 mM DTT. The protein was collected and concentrated by a Vivaspin 20 Centrifugal Concentrator. Then, the concentration of protein was determined by the Bradford Assay. The protein was flash frozen by liquid nitrogen and stored at −80 °C until needed for assays.

The protein expression of PRMT3, 5, and 8 was expressed as mentioned in a previous paper. PRMT3 and 8 were expressed and purified from *E. coli* by immobilized nickel chelating chromatography.^62^ 6× His-MEP50 was coexpressed with PRMT5 by the viral expression system, Bac-to-Bac baculovirus (Invitrogen, Life Technologies).^62^

### Peptide Synthesis

All of the peptides (Table 1) were synthesized using an automated AAPPTec Focus XC peptide synthesizer with the Fmoc solid-phase synthesis protocol and purified on reverse-phase C18 columns. The detailed protocols were described in our previously published papers.^14,61^

**Table 1:** Peptide Sequences

peptide	sequence
H4(1–20)-Biotin	SG**R**GKGGKGLGKGGAKRHRK-(Biotin)
Biotin-H4(1–22)	(Biotin)-SG**R**GKGGKGLGKGGAKRHRKVL
R4	Ac-GG**R**GGFGG**R**GGKGG**R**GGFGG**R**GGFG
H4(1–21)	Ac-SG**R**GKGGKGLGKGGAKRHRKV

### Radioactive Methyltransferase Assay

#### Scintillation Proximity Assay

All reactions were performed in 96-well plates at RT for 30 min and quenched with 30 μL of 100% isopropanol and an equal volume of the reaction mixture. The results were read by the MicroBeta 2450 Microplate Counter (PerkinElmer Revvity) after adding 10 μL of 20 mg mL^–1^ of streptavidin-coated beads to the reaction tube.

#### Single-Point Relative Activity Determination

The reaction consisted of 0.02 μM enzyme, 0.5 μM [^3^H]-SAM, and 1 μM H4(1–20)-Biotin in a 30 μL total reaction volume. The final reaction buffer concentration was 50 mM HEPES (pH 8), 10 mM NaCl, 0.5 mM EDTA, and 0.5 mM DTT.

The relative activity was calculated using eq 1:

1 $$\mathrm{relative}\;\mathrm{activity}=\frac{e\mathrm{xperimental}\;\mathrm{CPM}-n\mathrm{egative}\;c\mathrm{ontrol}\;\mathrm{CPM}}{p\mathrm{ositive}\;c\mathrm{ontrol}\;\mathrm{CPM}-n\mathrm{egative}\;c\mathrm{ontrol}\;\mathrm{CPM}}$$

where CPM is the counts per minute.

#### Activation by Small Molecules

The reaction consisted of 0.02 μM enzyme, 20 mM chemical molecule, 1 μM H4(1–20)-Biotin, and 0.5 μM [^3^H]-SAM in the final concentration of 50 mM HEPES (pH 8), 10 mM NaCl, 0.5 mM EDTA, and 0.5 mM DTT reaction buffer. The reaction was initiated with [^3^H]-SAM.

#### Concentration Dependence on 4-Methylimidazole

Various concentrations of 4-methylimidazole, 0.5–256 mM, were used. The reagents were input into the reaction tube in this specific order: 0.04 μM enzyme, various concentrations of 4-methylimidazole, 1 μM Biotin-H4(1–22), and then 0.5 μM [^3^H]-SAM to initiate the reaction. The reaction buffer consisted of 50 mM HEPES (pH 8), 10 mM NaCl, 0.5 mM EDTA, and 0.5 mM DTT.

#### Effect of 4-Methylimidazole on PRMT1-WT and PRMT1-H293G

The final concentration of PRMT1-WT or PRMT1-H293G was 0.04 μM, 20 mM 4-methylimidazole, 1 μM Biotin-H4(1–22), and 0.5 μM [^3^H]-SAM in 50 mM HEPES pH 8, 10 mM NaCl, 0.5 mM EDTA, and 0.5 mM DTT reaction buffer.

#### Enzyme Kinetics

The reaction mixture consisted of 0.04 μM PRMT1 or PRMT1-H293G, 20 mM 4-methylimidazole, 0.1, 0.15, 0.2, 0.25, 0.3, 0.4, 0.5, and 0.6 μM Biotin-H4(1–22), and 0.5 μM [^3^H]-SAM. The reaction buffer consisted of 50 mM HEPES (pH 8), 10 mM NaCl, 0.5 mM EDTA, and 0.5 mM DTT.

Kinetic analysis was performed using the Hill equation (eq 2):

2 $$\mathrm{rate}\;\left(\min^{-1}\right)=\frac{v}{\left[E\right]}=\frac{k_{\mathrm{cat}}{\left[S\right]}^{n}}{{K_{M}}^{n}+{\left[S\right]}^{n}}$$

#### pH Dependence

To test for pH dependence, reaction buffers with various pH levels were created. Initially, a 2× reaction buffer of 100 mM HEPES (pH 6.5, 7.0, 8.0, 8.5, or 9.0), 20 mM NaCl, 1 mM EDTA, and 1 mM DTT was used as the reaction buffer. All reaction reagents were prepared with the final concentration of 1× reaction buffer. The reaction mixture was added in the following order: 0.04 μM PRMT1-H293G, 20 mM 4-methylimidazole, 0.5 μM Biotin-H4(1–22), and then 0.5 μM [^3^H]-SAM.

### Filter Binding Assay

Each reaction contained 0.04 μM enzyme, 20 mM 4-methylimidazole, 1 μM peptide, and 0.5 μM [^3^H]-SAM in the reaction buffer, which consisted of 50 mM HEPES (pH 8), 10 mM NaCl, 0.5 mM EDTA, and 0.5 mM DTT. The reaction was at RT for 30 min and quenched with an equal reaction volume of 100% isopropanol. The reaction solution was transferred to phosphocellulose P81 filter paper (2.2 × 2.2 cm, Protein Chemistry & Metabolism Unit at Vincent’s Institute of Medical Research) and air-dried for 30 min. Afterward, the filter paper was washed with 50 mM NaHCO3 (pH 9.0) for 3 times (20 min per wash). The filter paper was then air-dried overnight. The next day, the filter paper was placed in a scintillation vial and soaked in 5 mL of the scintillation cocktail (Ultima Gold mixture, PerkinElmer) to measure the counts per minute (CPM) using the Beckman Coulter LS6500.

### MALDI-MS of Peptide Methylation

The reaction was conducted with 50 mM HEPES (pH 8), 10 mM NaCl, 0.5 mM EDTA, and 0.5 mM DTT reaction buffer. PRMT1-H293G was incubated with 4-methylimidazole for 30 min on ice at concentrations of 1 μM and 20 mM, respectively. After the incubation, 10 μM Ac–H4(1–21) was added to the reaction mixture. Lastly, 50 μM SAM was added to the reaction mixture to initiate the reaction for 2 h in a 25 °C water bath. The reaction was quenched with 20 μL of 5% TFA and sent to MALDI-MS.

The experimental procedure for R4 peptide methylation by MALDI-MS was the same as that of Ac–H4(1–21). However, the concentrations of the reagents were 0.5 μM PRMT1-H293G, 20 mM 4-methylimidazole, 5 μM R4 peptide, and 50 μM SAM. The reaction time was 30 min in a 25 °C water bath.

### Methylation of Hypomethylated HeLa Cell Lysate

HeLa cells were grown in DMEM with 10% FBS and 1% penicillin–streptomycin. After the confluency reached approximately 80%, HeLa cells were treated with 20 μM adenosine dialdehyde for 24 h. The cell lysate was extracted with M-PER Mammalian Protein Extraction Reagent and the protease cocktail inhibitor (100:1 dilution). The mixture was sonicated using a Qsonica instrument, and the concentration was checked by the Bradford Assay. The final concentration of reaction buffer was 50 mM HEPES (pH 8), 10 mM NaCl, 0.5 mM EDTA, and 0.5 mM DTT. Recombinant PRMT1-H293G or PRMT1 was incubated on ice with 4-methylimidazole for 30 min. The concentration of the enzyme was 1 μM. The concentrations of 4-methylimidazole were 5, 10, and 20 mM. Afterward, 15 μg of cell lysate and 50 μM SAM were added to the reaction. The reaction was completed at 30 °C for 3 h, and 5× protein loading buffer (National Diagnostics) was added to halt the reaction. The reaction was heated for 10 min at 95 °C and resolved on 12% SDS-PAGE. The ADMA detection was completed by Western blotting with an ADMA primary antibody (1:2000 dilution, a gift from Dr. Mark T. Bedford) and an anti-rabbit IgG HRP-linked secondary antibody (1:3000 dilution, Cell Signaling Technology).^63^ The nitrocellulose membrane was stripped with a mild-stripping buffer and reprobed with β-Actin. The recipe of the membrane mild-stripping buffer consisted of 1.5 g of glycine, 0.1 g of SDS, and 1 mL of Tween 20 for a 100 mL volume. The buffer was adjusted to pH 2.2. The membrane was stripped with membrane stripping buffer twice for 10 min each time. The membrane was soaked in PBS twice for 10 min and then in TBST twice for 5 min. The membrane was blocked with 5% skim milk for 1 h before incubating with the antibody. β-Actin detection consisted of β-Actin (C4) sc-47778 (1:1000, Santa Cruz Biotechnology) for the primary antibody and the goat anti-mouse IgG HRP conjugate (1:3000, TONBO Biosciences) for the secondary antibody.

## Data Availability

All data are included in the manuscript. Any additional data that support the findings of this study are available from the corresponding author upon reasonable request.
